# Identifying Major Drivers of Antioxidant Activities in Complex Polyphenol Mixtures from Grape Canes

**DOI:** 10.3390/molecules27134029

**Published:** 2022-06-23

**Authors:** Manon Ferrier, Kévin Billet, Samantha Drouet, Duangjai Tungmunnithum, Magdalena Anna Malinowska, Cécile Marchal, Sandrine Dedet, Nathalie Giglioli-Guivarc’h, Christophe Hano, Arnaud Lanoue

**Affiliations:** 1 EA 2106 Biomolécules et Biotechnologies Végétales, UFR des Sciences Pharmaceutiques, Université de Tours, 31 Av. Monge, 37200 Tours, France; manon.ferrier@univ-tours.fr (M.F.); kevin.billet@univ-tours.fr (K.B.); magdalena.malinowska@pk.edu.pl (M.A.M.); nathalie.guivarch@univ-tours.fr (N.G.-G.); 2 Laboratoire de Biologie des Ligneux et des Grandes Cultures (LBLGC), INRA USC1328 Université d’Orléans, CEDEX 2, 45067 Orléans, France; samantha.drouet@univ-orleans.fr (S.D.); duangjai.tun@mahidol.ac.th (D.T.); hano@univ-orleans.fr (C.H.); 3 Department of Pharmaceutical Botany, Faculty of Pharmacy, Mahidol University, Bangkok 10400, Thailand; 4 Faculty of Chemical Engineering and Technology, Cracow University of Technology, 24 Warszawska St., 31-155 Cracow, Poland; 5 Grapevine Biological Resources Center, INRAE, Unité Expérimentale Domaine de Vassal, University of Montpellier, 34340 Marseillan, France; cecile.marchal@inrae.fr (C.M.); sandrine.dedet@inrae.fr (S.D.)

**Keywords:** grape canes, metabolomics, polyphenols, antioxidant activities, grape varieties

## Abstract

Grape canes represent a valuable source of numerous polyphenols with antioxidant properties, whose compositions vary depending on the genotype and environmental factors. Antioxidant activities of pure molecules are often reported without considering possible interactions that may occur in complex polyphenol mixture. Using UPLC-MS-based metabolomics and unsupervised classification, we explored the polyphenol variations in grape cane extracts from a collection of European varieties. Antioxidant activities were assessed using ORAC, ABTS, DPPH, FRAP, CUPRAC and chelation assays. Pairwise correlations between polyphenols and antioxidant capacities were performed to identify molecules that contributed more to the antioxidant capacities within a complex mixture of polyphenols.

## 1. Introduction

*Vitis vinifera* L. produces various antioxidant polyphenols, including stilbenoids that preferentially accumulate in woody tissues [1,2]. Large amounts of grape woods are pruned during winter, with a small part being recycled for composting, while added-value biomolecules could be valorized from this biomass. Consequently, a sustainable circular economy has been proposed based on the reuse of grape canes as a new source of dietary polyphenols to promote health benefits via antioxidant properties [3,4,5]. *E*-resveratrol, the “flagship molecule” from grapevine, exhibits antidiabetic, antitumor, anti-inflammatory, antiaging and neuroprotective activities [6,7,8], and *E*-piceatannol, an hydroxylated analogue of resveratrol, shows antioxidant, anti-inflammatory, antimutagenic, anticancer as well as antimicrobial activities [9,10]. Whereas *E*-resveratrol antioxidant activity has been extensively investigated, the biological activities and potential health benefits of other compounds found in grape cane extracts such as *E*-resveratrol-oligomers have also been recently reported on [11,12,13,14].

In grape cane extracts, a set of ten molecules constitutes the most abundant polyphenols, including *E*-ε-viniferin, *E*-resveratrol, ampelopsin A, catechin, hopeaphenol, epicatechin, *Z*/*E*-vitisin B, *E*-piceatannol, *E*-miyabenol C and isohopeaphenol. Nevertheless, the absolute concentrations of these major compounds were found to be highly variable depending on the considered varieties, but also due to environmental conditions [15,16,17]. Note that the accurate quantification of stilbenoids requires calibration curves created with the pure molecule, whereas expression as a resveratrol equivalent leads to their underestimation [16,18]. Additionally, metabolomics profiling studies showed that the chemodiversity of grape cane extracts is much broader and complex. It, at least, includes more than 40 polyphenols related to phenolic acids, flavonoids, procyanidins and stilbenoids as resveratrol oligomers (degrees of oligomerization 1–4). This cocktail of polyphenols with varying proportions forms the chemotype of a grape variety and could likely influence the total antioxidant capacity of the extract [5,19].

Grape cane extracts used as a complex mixture of polyphenols have been reported to scavenge the ABTS free radical cation and to enhance human glutathione peroxidase and superoxide dismutase [20]. Moreover, when grape cane extracts were given as food supplements to hamsters fed with a hyperlipidic diet, a cardiovascular protection on early atherosclerosis was observed [21]. Interestingly, grape cane extracts showed enhanced antiproliferative effects and anti-inflammatory activities compared to *E*-resveratrol or *E*-ε-viniferin tested alone [22,23]. It has been reported that grape extracts should be considered a combination of numerous polyphenols exhibiting possible synergistic/additive/antagonistic interactions [24]. Indeed, the combination of *E*-resveratrol and oxyresveratrol showed synergistic antioxidant activities and the fractionation of grape cane extracts revealed different antioxidant capacities compared to the single molecules [25,26].

Several in vitro techniques are classically used to identify antioxidant activities. Antioxidants can neutralize reactive oxygen species with several mechanisms, including hydrogen atom transfer (HAT), single-electron transfer (SET) and the ability to chelate transition metals [27]. The oxygen radical antioxidant capacity (ORAC) is known as a HAT-based assay. SET assays encompassed cupric ion antioxidant capacity (CUPRAC), ferric-reducing antioxidant power (FRAP) and a scavenging effect in relation to 1,1-diphenyl-2-picrylhydrazyl (DPPH) and 2,2′-azino-bis(3-ethylbenzothiazoline-6-sulphonic acid (ABTS). Despite ABTS and DPPH usually being classified as SETs, they can also react through HAT and are considered as mixed-mode reactions [27]. Aside from these two mechanisms for radical scavenging actions, transient metal ion chelation is also considered as an antioxidant mechanism based on its capacity to inhibit the Fenton reaction [28].

The scavenging ability of an individual metabolite is related to the structure, but also to the influence of the other compounds exhibiting biological activity. Quantitative structure–activity relationship (QSAR) investigations determined the influence of compound topology, resonance stabilization and intramolecular hydrogen bonding [29]. Scavenging activity related to the compound structure depends on the number and the position of the hydroxyl groups. A QSAR study on several stilbenoids showed that scavenging capacities of stilbenoids depend on both HAT and SET mechanisms [30].

This study aims to identify the main drivers of antioxidant activities in polyphenol-rich grape cane extracts in European varieties. We performed the metabolic profiling of grape cane extracts from a large germplasm collection of European varieties, as well as the assessment of antioxidant capacities of polyphenol-rich and -poor grape cane extracts. We used a complete set of in vitro antioxidant tests based on complementary mechanisms, namely SET, HAT and mixed HAT/SET modes or chelating capacity. Finally, we explored the correlations between complex polyphenol contents and antioxidant capacities.

## 2. Results

### 2.1. Metabolic Profiling and Unsupervised Classification of Grape Cane Extracts from European Varieties

From the thousands of existing European grape varieties, we used 44 grape varieties previously selected for their distribution among genetic clusters based on simple sequence repeats (SSR) [5]. A complete grape cane metabolic profile was performed on each grape variety in both ESI+ and ESI− modes using the molecular ion list previously described [19]. Over 42 detected analytes, 20 compounds were unambiguously identified through external standards and 22 were tentatively assigned according to the elution order, UV spectra and MS data from the literature (Table S1, Supplementary Materials).

**Figure 1:**
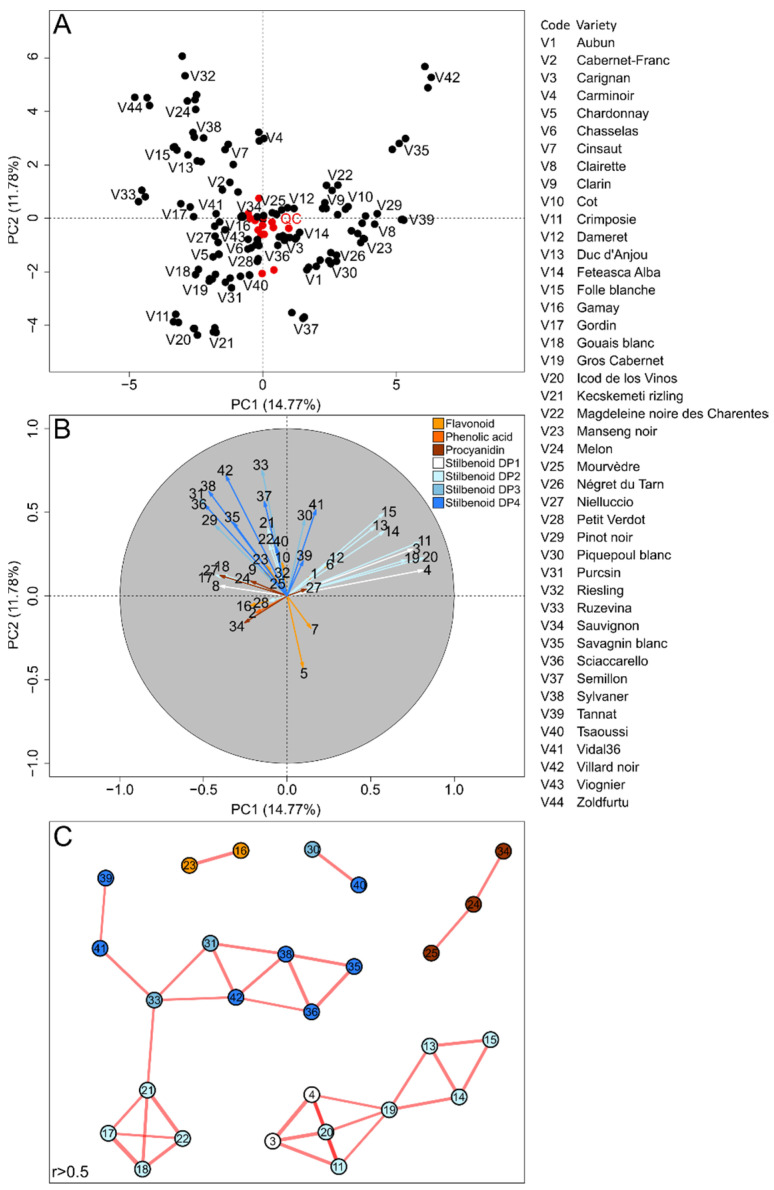
Unsupervised classification using PCA performed on the metabolomics profiles of 44 European grape varieties. Score plot (**A**) on samples (dark) and QC (red). Loading plot (**B**) with variables colored according to the polyphenol class and numbered according to metabolite names given in the Section 3. Spearman correlation-based networks of polyphenol metabolism (**C**), node numbers represent the metabolites and the color refers to the polyphenol class. Short edge distances indicate the Spearman coefficient importance and the correlation significance.

We assessed the polyphenol metabolic variability through a principal component analysis (PCA) performed on the relative abundance of the 42 metabolites within the 44 selected European varieties (Figure 1A,B).

The PCA score plot explained 26.55% of the variation of the two first principal components (PC) and presented grouped and centered quality control (QC) observations, thus, validating the robustness of metabolomics analyses (Figure 1A). On the loading plot (Figure 1B), the variables of stilbenoid monomers (DP1) and dimers (DP2) were projected together in PC1- and PC2-positive scores, whereas stilbenoid trimers (DP3) and tetramers (DP4) were projected together in PC1-negative and PC2-positive scores. Other polyphenol classes corresponding to phenolic acids, flavonoids and procyanidins weakly correlated with PC1 and PC2, showing a poor contribution of these compounds to varietal metabotypes (Figure 1B). The present PCA was used for the rapid identification of grape varieties with interesting polyphenol metabotypes. Grape varieties with high contents in both stilbenoids DP1-2 and DP3-4 were projected in PC1- and PC2-positive scores, namely, Savagnin blanc (V35) and Villard noir (V42), French–American hybrid cultivars. Guerrero et al. (2016) [17] previously observed that Villard noir was particularly rich in *E*-ε-viniferin (DP2) and hopeaphenol (DP4). Grape varieties rich only in stilbenoids DP3 and DP4 were projected in PC1-negative and PC2-positive scores, such as Riesling (V32). Interestingly, Lambert et al. (2013) [16] previously described Riesling as a stilbenoid DP4-rich variety. Grape varieties rich only in stilbenoid DP1 and DP2 were projected in a PC1-positive score, such as Pinot noir (V29). Such levels of stilbenoid DP1 and DP2 were already mentioned for Pinot noir [16]. Conversely, varieties with a generally poor accumulation of stilbenoids, such as Crimposie (V11), Icod de los Vinos (V20) and Kecskemeti Rizling (V21), were projected in PC1- and PC2-negative scores.

In addition, a covariation metabolite network was generated using the Spearman correlation R > 0.5 and *p*-value < 0.05 (Figure 1C). The network showed clusters corresponding to different subclasses of grape polyphenols, including procyanidins, flavonoids, stilbenoids DP1-2 and stilbenoids DP3-4. The network topology showed that structurally related compounds were accumulated together in the specific grape varieties, confirming previous observations on a smaller variety collection [19]. Despite poor knowledge on stilbenoid oligomerization pathways [31,32], the present clustering showed that stilbenoid DP1-2 shared common biosynthetic regulation and also stilbenoid DP3-4.

Finally, to estimate the plasticity of polyphenol metabolism, we calculated the coefficient of variation for the 42 metabolites among the 44 selected grape varieties (Figure S1). The total amount of polyphenol showed only weak variations (17%) confirming previous observations on the total polyphenol content of grape canes [18]. However, the specific composition in polyphenols appeared highly variable. Indeed, most stilbenoids presented variation coefficients higher than 50%, reaching 141% and 169% for α-viniferin and restrytisol 3, respectively. Such stilbenoids with strong variation coefficients might be considered as potential varietal biomarkers, whereas procyanidins presenting weak variations (25%) are most likely accumulated at constitutive levels regardless of the variety.

### 2.2. Antioxidant Capacities of Grape Cane Extracts

We performed a polyphenol enrichment through a reflux extraction of the ten varieties representing the full amplitude of polyphenol variations among the 44 selected European varieties [5], namely; Villard noir (V42), Savagnin blanc (V35), Riesling (V32), Magdeleine noire des Charentes (V22), Piquepoul blanc (V30), Sauvignon (V34), Sémillon (V37), Kecskemeti Rizling (V21), Tsaoussi (V40) and the Icod de los Vinos (V20). These polyphenol-enriched grape cane extracts were assessed for FRAP, CUPRAC, Chelation, ABTS, DPPH and ORAC antioxidant activities (Table 1). Results highlighted a strong varietal effect (*p* < 0.001) for each antioxidant test. Savagnin presented the highest FRAP and CUPRAC activities when Sauvignon presented the lowest. Savagnin blanc (V35) and Villard noir (V42) showed high capacities for ORAC, ABTS and DPPH activities. The ORAC capacities of these grape cane extracts were higher than the values reported for 30 other plant extracts [33], highlighting the great potential of grape wood biomass to be valorized as a food supplement with antioxidant properties. Note that the ORAC capacities of grape cane extracts could be further increased with polyphenol fractionation using centrifugal partition chromatography [25].

**Figure 2:**
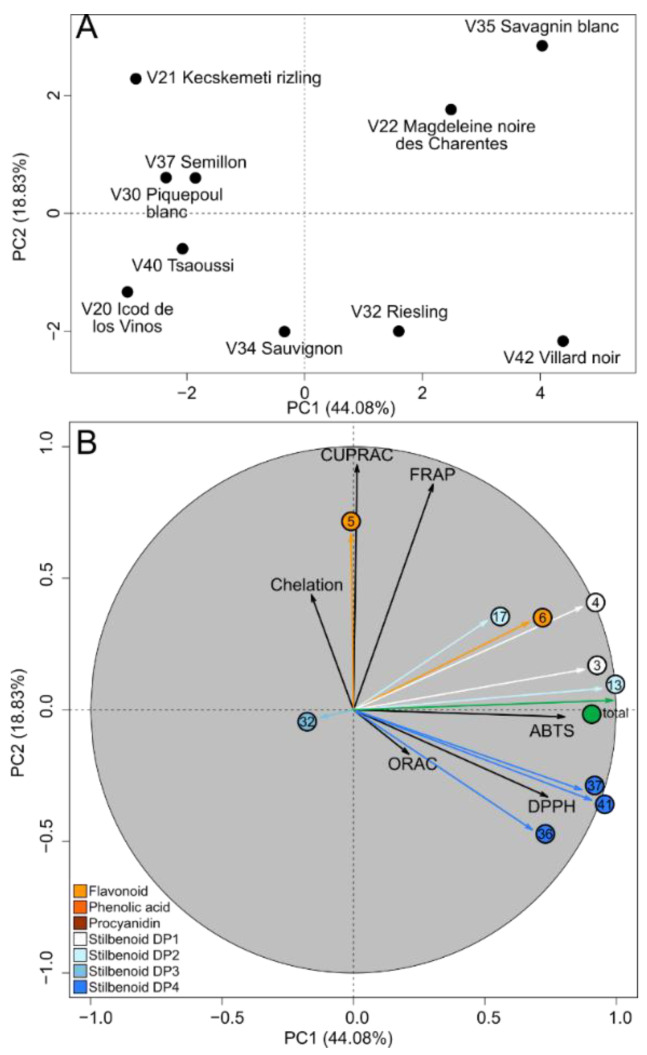
Unsupervised classification using PCA performed on polyphenol-enriched extracts for both antioxidant capacities and polyphenol contents. Score plot (**A**). Loading plot (**B**) with variables colored according to polyphenol class.

To identify specific polyphenols driving antioxidant capacities in grape cane extracts, we performed a PCA (Figure 2) on 17 variables, including major polyphenols and antioxidant capacities using six antioxidant tests.

**Figure 3:**
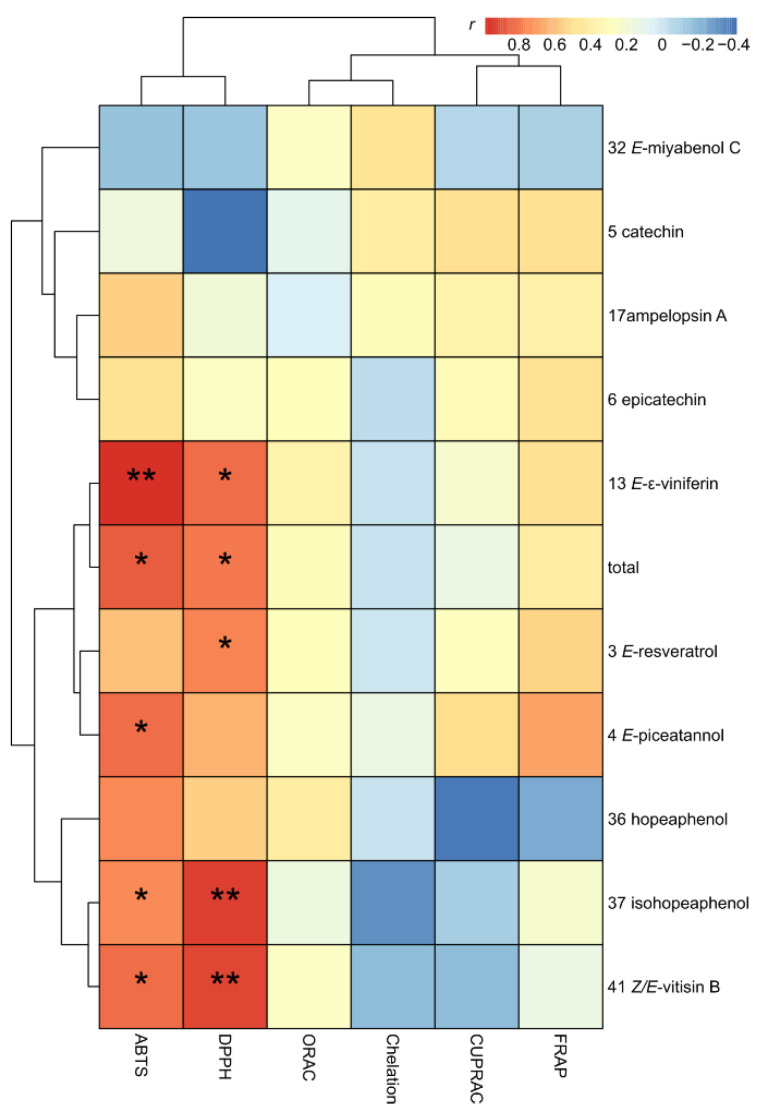
Pairwise Spearman correlations of single-metabolite and antioxidants tests (R; * *p* < 0.05 and ** *p* < 0.01).

The first two PCs explained 62.91% of the initial variance. Varieties with both high polyphenol contents and high ABTS and DPPH capacities were projected in a PC1-positive score (Savagnin blanc (V35), Villard noir (V42) and Magdeleine noire des Charentes (V22)), whereas polyphenol-poor varieties associated with low ABTS and DPPH capacities were projected in a PC1-negative score (Icod de los Vinos (V20) Tsaoussi (V40) and Kecskemeti Rizling (V21)). Varieties with high CUPRAC and FRAP properties (Savagnin blanc (V35), Kecskemeti Rizling (V21) and Magdeleine noire des Charentes (V22)) were projected in a PC2-positive score. Although weakly projected on PC, iron-chelation seemed to be grouped with FRAP and CUPRAC, whereas ORAC seemed to be related to ABTS and DPPH (Figure 2B). The ORAC capacity, based on the HAT mechanism, was projected on a PC2-positive score with stilbenoid DP4. These results were consistent with computational studies showing that the OH groups in the trans-stilbene moiety presented a predominant HAT capacity [30], whereas most polyphenols were projected in a PC1-positive score with ABTS and DPPH, and catechin (5) was projected in a PC2-positive score with FRAP and CUPRAC. The present results suggested that in a complex mixture of polyphenols, several compounds mainly drive ABTS and DPPH antioxidant capacities, while others drive CUPRAC and FRAP antioxidant capacities. Then, we used pairwise Spearman correlations to investigate the relationship between relative levels of single polyphenols and antioxidant capacities (Figure 3).

The heatmap clearly discriminated two clusters of polyphenols. The first group, including *E*-resveratrol (3), *E*-piceatannol (4), *E*-ε-viniferin (13), hopeaphenol (36), isohopeaphenol (37), *Z*/*E*-vitisin B (41) and total polyphenols, was positively and significantly correlated (r > 0.5; *p* < 0.05) with ABTS and DPPH capacities using two antioxidant tests based on mixed-mode (HAT/SET) antioxidant ability. The present results were consistent with a study on QSAR created for several stilbenoids, showing that the scavenging capacity of these compounds depends on both HAT and SET mechanisms [30]. Interestingly, in a study analyzing grape juices from 11 varieties produced in Brazil, the resveratrol content was also strongly correlated with DPPH and ABTS activities [34]. A second group, including, catechin (5), epicatechin (6), ampelopsin A (17) and *E*-miyabenol C (32), was weakly or not at all correlated with antioxidant capacities. We found that molecules driving the total antioxidant capacity in polyphenol mixtures, such as grape cane extracts, were not those with the highest antioxidant capacities such as a pure molecule [25]. Only a few studies investigated the variation of single compounds within a complex mixture in relation with antioxidant capacities, and combination studies usually included only several compounds [26]. However, several reports showed that grape antioxidants used as a polyphenol combination might potentiate chemoprevention efficacy [24,35,36].

## 3. Materials and Methods

### 3.1. Plant Materials

The experiments were performed on 44 *V. vinifera* L. healthy cultivars available from the INRA grape repository at “Domaine de Vassal” (34340 Marseillan-Plage, France; https://www6.montpellier.inra.fr/vassal/; accessed date 3 June 2022). We selected the cv. Aubun (V1), Cabernet Franc (V2), Carignan (V3), Carminoir (V4), Chardonnay (V5), Chasselas (V6), Cinsaut (V7), Clairette (V8), Clarin (V9), Cot (V10), Crimposie (V11), Dameret (V12), Duc d’Anjou (V13), Feteasca Alba (V14), Folle blanche (V15), Gamay (V16), Gordin (V17), Gouais blanc (V18), Gros Cabernet (V19), Icod de los Vinos (V20), Kecskemeti Rizling (V21), Magdeleine noire des Charentes (V22), Manseng noir (V23), Melon (V24), Mourvèdre (V25), Négret du Tarn (V26), Nielluccio (V27), Petit Verdot (V28), Pinot noir (V29), Piquepoul blanc (V30), Purcsin (V31), Riesling (V32), Ruzevina (V33), Sauvignon (V34), Savagnin blanc (V35), Sciaccarello (V36), Sémillon (V37), Sylvaner (V38), Tannat (V39), Tsaoussi (V40), Vidal 36 (V41), Villard noir (V42), Viognier (V43) and Zoldfürtü (V44). For each variety, we harvested five grape canes on each of the five collected vines in January, 2016. The grape cane storage was performed as previously described [18]. Afterwards, cane samples were ground twice and the stilbenoids were extracted from the powder as previously described [37].

### 3.2. Chemicals

Acetonitrile and ethanol were purchased from Thermo Fisher Scientific (Courtaboeuf, France). A Millipore Milli-Q system (Merck Millipore, Molsheim, Germany) provided ultrapure water. Gallic acid, caffeic acid, *E*-Resveratrol, *E*-piceatannol, catechin, epicatechin, gallocatechin, *E*-piceid, astilbin, quercetin-3-*O*-glucuronide, procyanidin B1 and procyanidin B2 were obtained from Sigma-Aldrich (St. Louis, MO, USA); *E*-ε-viniferin and quercetin-3-*O*-glucoside were purchased from Extrasynthèse (Genay, France) and *E*-miyabenol C from Ambinter (Paris, France). Pallidol and *E*-δ-viniferin were graciously provided by Mary-Lorène Goddard (EA-3991, Université de Haute-Alsace, Colmar, France). *Z*/*E*-vitisin B, ampelopsin A and hopeaphenol were purified from grape canes as previously described [37]. The 2,2′-Azo-bis-(2-amidinopropane) hydrochloride (ABAP), fluorescein, 6-hydroxy-2,5,7,8-tetramethylchroman-2-carboxylic acid (Trolox), 2,2′-azino-bis(3-ethylbenzothiazoline-6-sulphonic acid (ABTS), potassium persulfate, 2,2-diphenyl-1-picrylhydrazyl (DPPH), 2,4,6-Tri(2-pyridyl)-s-triazine (TPTZ), Iron(III) chloride (FeCl_3_), copper(II) chloride (CuCl_2_), 2,9-Dimethyl-1,10-phenanthroline (neocuproine) and 3-(2-Pyridyl)-5,6-diphenyl-1,2,4-triazine-p,p′-disulfonic acid monosodium salt hydrate (ferrozine) were obtained from (Sigma-Aldrich, Saint-Quentin Fallavier, France).

### 3.3. High-Performance Liquid Chromatography Analysis (HPLC-DAD)

An HPLC-DAD analysis was the same as previously reported [5]. Absolute quantifications were conducted using pure standards of catechin (5), epicatechin (6), *E*-Resveratrol (3), *E*-piceatannol (4), *E*-ϵ-viniferin (13), *E*-miyabenol C (32), ampelopsin A (17), *Z*/*E*-vitisin B (41) and hopeaphenol (36) using a five-point calibration curve (0-100 ppm). Quantification analyses were performed in triplicates using the Maxplot detection mode.

### 3.4. Ultra-High-Performance Liquid Chromatography Coupled to Mass Spectrometry (UPLC-MS)

The UPLC-MS system was composed of an ACQUITY™ Ultra Performance Liquid Chromatography system coupled to a photo diode array detector (PDA) and a Xevo TQD mass spectrometer (Waters, Milford, MA, USA) equipped with an electrospray ionization (ESI) source controlled by Masslynx 4.1 software (Waters, Milford, MA). A Waters Acquity HSS T3 C18 column (150 × 2.1 mm, 1.8 μm) achieved the analytes separation with a flow rate of 0.4 mL min^−1^ at 55 °C. The injection volume was 5 μL. The mobile phase was composed of 0.1% formic acid in water (solvent A) and 0.1% formic acid in acetonitrile (solvent B). The chromatographic separation was performed using an 18 min linear gradient from 5 to 60% solvent B, followed by washing and column reconditioning for 8 min. MS detection was performed in both positive and negative modes. The capillary voltage was 3,000 V and sample cone voltages were 30 and 60 V. The cone and desolvation gas flow rates were 60 and 800 Lh^−1^, respectively.

The analyte identification was performed as previously described [19]. Briefly, retention times, *m*/*z* values and UV spectra were compared to pure standards or data from literature when no authentic standards were available (Table S1). Gallic acid (1), caffeic acid (2), *E*-resveratrol (3), *E*-piceatannol (4), catechin (5), epicatechin (6), gallocatechin (7), *E*-piceid (8), astilbin (10), pallidol (11), *E*-ε-viniferin (13), *E*-δ-viniferin (15), quercetin-3-*O*-glucoside (16), ampelopsin A (17), quercetin-3-*O*-glucuronide (23), procyanidin B1 (24), procyanidin B2 (25), *E*-miyabenol C (32), hopeaphenol (36), isohopeaphenol (37) and *Z*/*E*-vitisin B (41) were identified by comparison with external standards. Peak nine gave an [M − H]^−^ ion at *m*/*z* 441 and formed daughter fragments at *m*/*z* 289 (catechin unit) and *m*/*z* 169 (gallic acid unit). This signature of peak nine was tentatively assigned to epicatechin 3-*O*-gallate 4. Peak 12 giving an [M − H]^−^ at *m*/*z* 453 was partially annotated as a *Z*-resveratrol dimer through its fragment at *m*/*z* 265 [38]. Peak 14 giving an [M − H]^−^ at *m*/*z* 453 was annotated as *E*-ω-viniferin through a comparison of its elution order with *E*-ε-viniferin (13) and *E*-δ-viniferin (15) [38,39,40]. Both peaks 18 and 19 with an [M − H]^−^ ion at *m*/*z* 469 were eluted after ampelopsin A (17) and were tentatively identified as structural isomers of scirpusin A [41,42]. Three peaks at *m*/*z* 471 (peaks 20–22) were putatively assigned to the oxidized resveratrol–resveratrol homodimers restrytisol A and B, and to an additional derivative [39,43]. Four compounds with an [M − H]^−^ ion at *m*/*z* 577 (peaks 24–27) were attributed to procyanidin B-types 4 and confirmed by the two standards of procyanidin B1 and B2 (peak 24 and 25). Peak 28 showed an [M − H]^−^ ion at *m*/*z* 615 and gave a product ion at the *m*/*z* 453 characteristic of hexose loss (162 Da) and was attributed to resveratrol dimer-glycoside [41]. Peak 29 with an [M − H]^−^ ion at *m*/*z* 677 was temporary assigned to α-viniferin [44]. Peaks 30, 31 and 33 presented [M − H]^−^ ions at *m*/*z* 679, such as *E*-miyabenol C (peak 32), and were annotated as resveratrol trimers. Peak 34 presented an [M − H]^−^ ion at *m*/*z* 865, producing daughter ions at *m*/*z* 576 (loss of catechin) and *m*/*z* 289 (catechin unit), which we assigned as a procyanidin trimer [45]. According to Ito et al. (1999), we temporarily assigned peak 35 with an [M − H]^−^ ion at *m*/*z* 904 to a dehydrogenated resveratrol tetramer [46]. Several compounds exhibiting [M − H]^−^ ions at *m*/*z* 905 (peaks 38–40), such as hopeaphenol (36), isohopeaphenol (37) and *Z*/*E*-vitisin B (41), were assigned to resveratrol tetramers [38,47]. Finally, peak 42, producing an [M − H]^−^ ion at *m*/*z* 923, was assigned to viniferol E [48].

Extraction and UPLC-MS analyses were performed in triplicates. A selected ion monitoring (SIM) mode and the resulting SIM chromatograms were integrated using the ApexTrack algorithm with a mass window of 0.1 Da and a relative retention time window of 0.2 min, followed by Savitzky–Golay smoothing (iteration = 1; width = 1) using Targetlynx software. The resulting pairs of *m*/*z* values and retention times were visually controlled. The robustness of measurements and analytical variability were evaluated through quality control (QC) injections. Ten QCs were injected before the batch, one QC was injected into each eight samples during the batch and, finally, ten QC were injected after the batch. A principal component analysis (PCA) was employed to evaluate the reproducibility of the UPLC-MS method [49]. The robustness of measurements and analytical variability was evaluated through quality control (QC) injections, as recommended by Fiehn et al. [49].

### 3.5. Polyphenol-Enriched Extracts and Antioxidant Tests

#### 3.5.1. Extraction

One-gram of dried ground stems was extracted with 20 mL of ethanol/water mixture (60/40; *v*/*v*). Samples were extracted for 30 min under sonication at 83 °C (Al04-04, Advantage Lab, Darmstadt, Germany) and centrifuged at 13,000 rpm for 10 min. Then, supernatants were evaporated using a Heidolph 94,200 Bioblock rotavapor (Schwabach, Germany) coupled with a Vacuubrand PC500 series pump (Wertheim, Germany). The resulting extracts were then freeze-dried, allowing for stilbenoid-enriched extracts. The extraction yields ranged between 5 and 10% (data not shown).

#### 3.5.2. Determination of Oxygen Radical Antioxidant Capacity (ORAC)

ORAC assays were adapted from Prior et al. (2003) [50]. We mixed 10 µL of grape stem extracts with 190 µL of fluorescein (0.96 µM) in 75 mM phosphate buffer pH 7.4. Mixtures were incubated for at least 20 min at 37 °C with intermittent shaking. Then, we added 20 µL of 119.4 mM 2,2′-Azo-bis-(2-amidinopropane) hydrochloride (ABAP) and read the fluorescence intensity every 5 min for 2.5 h at 37 °C using a fluorescence spectrophotometer (Bio-Rad, Marnes-la-Coquette, France) set with 485 nm and 535 nm as respective excitation and emission wavelengths. Assays were conducted in triplicate and we expressed antioxidant capacity as 6-hydroxy-2,5,7,8-tetramethylchroman-2-carboxylic acid (Trolox) equivalent antioxidant capacity (TEAC). The Trolox value was equivalent to a 1 mM solution of the tested antioxidant (Trolox, range = 12.5–100 µM, r^2^ = 0.9998).

#### 3.5.3. Determination of the Free Radical Scavenging Capacity through ABTS

The free radical scavenging capacity determination of the grape stem extracts was adapted from Re et al. (1999) [51] using the ABTS+• radical cation decolorization assay. ABTS solution was prepared at 7 mM in Milli-Q water. The ABTS+• radical cation was produced by reacting the ABTS solution with a 2.45 mM final concentration of potassium persulfate. The mixture was incubated in the dark at 25 °C for 12–16 h. The ABTS+• solution was then diluted to an absorbance of 0.7 at 734 nm. After the addition of 100 µL of grape stem extract to 3 mL of ABTS+•, the absorbance was read after 10 min at 30 °C. All solutions were used on the day of preparation. Assays were created in triplicate and the free radical scavenging capacity was expressed as TEAC (Trolox, range = 12.5–250 µM, r^2^ = 0.9987).

#### 3.5.4. Determination of the DPPH Scavenging Capacity

The ability of grape stem extracts to scavenge the DPPH radical was measured as described by Brand-Williams et al. (1995) [52]. Aliquots (20 µL) of sample extract were prepared at 1 mg mL^−1^, mixed with 3 mL of 6 × 10^−5^ M ethanolic DPPH solution and incubated in the dark at 25 °C for 90 min. The absorbance of the resulting mixture was measured at 515 nm. Grape stem extract antioxidant capacities were then expressed as Trolox equivalent antioxidant capacity per gram of dry extract using an external calibration (Trolox, range = 12.5–250 µM, r^2^ = 0.9994).

#### 3.5.5. Determination of the Ferric-Reducing Antioxidant Power (FRAP)

FRAP measurement was adapted from Benzie and Strain (1996) [53]. We mixed 10 µL of grape stem extracts with 190 µL of FRAP (10 mM TPTZ; 20 mM FeCl_3_•6H_2_O; 300 mM acetate buffer pH 3.6; ratio 1:1:10 (*v*/*v*/*v*)). Mixtures were incubated for 15 min at 25 °C and absorbance was read at 630 nm (BioTek ELX800 Absorbance Microplate Reader, Thermo Fisher Scientific, Villebon-sur-Yvette, France). Assays were conducted in triplicates and data were expressed as TEAC (Trolox, range = 12.5–250 µM, r^2^ = 0.9981).

#### 3.5.6. Determination of the Cupric Ion-Reducing Antioxidant Capacity (CUPRAC)

The evaluation of the cupric ion-reducing antioxidant capacity (CUPRAC) of grape stem extracts was adapted from Apak et al. (2007) [54]. We mixed 10 µL of grape stem extracts with 190 µL of a 1 × 10^−2^ M copper (II) chloride solution (CuCl_2_•2H_2_O; ammonium acetate buffer pH7.0; 7.5 × 10^−3^ M neocuproine (2,9-dimethyl-1,10-phenanthroline) in ethanol; 1:1:1, *v*:*v*:*v*) and read absorbance at 450 nm after 30 min. The grape stem extract CUPRAC antioxidant capacities were expressed as TEAC (Trolox, range = 12.5–250 µM, r^2^ = 0.9997).

#### 3.5.7. Determination of Iron-Chelating Capacity

The iron-chelating capacity determination was adapted from Mladěnka et al. (2011) [55]. We mixed 10 µL of grape stem extracts with a ferrous iron solution at 50 µM final concentration in HEPES (pH 6.8) buffer and 50 µL ferrozine (5 mM aqueous solution). Experiments were conducted in 96-well microplates. Each extract was measured with and without (blank) ferrozine. We read the absorbance at 550 nm immediately after ferrozine addition and 5 min later (BioTek ELX800 Absorbance Microplate Reader, Thermo Fisher Scientific, Villebon-sur-Yvette, France). Chelating values were expressed in µM of fixed iron.

### 3.6. Statistical Analysis

Data were analyzed with R (v3.4.2; R Core Team, 2017). Principal component analyses were performed using the “FactoMineR” package [56]. Significance of average difference among extract contents was evaluated with Kruskal–Wallis one-way ANOVA and followed by Fisher’s least significant difference (*p* < 0.05) as post hoc test. Pairwise correlations within the HPLC data matrix and antioxidant test results were calculated for all the 66 pairs. Matrices of pairwise Spearman correlation coefficients were calculated in R with the “cor” function and significance of association was obtained with the “cor.test” function. Significant correlations (R > 0.5 and *p* < 0.05) were visualized using the “pheatmap” package [57]. Hierarchical cluster analyses (HCA) using Ward’s minimum variance method were performed on SSR data using SIMCA P+ version 12.0 (Umetrics AB, Umeå, Sweden).

## 4. Conclusions

UPLC-MS-based metabolomic screening coupled with unsupervised classification was used to identify specific polyphenol signatures among grape cane extracts from European varieties. Interestingly, Savagnin Blanc and Villard noir presented peculiar polyphenol metabotypes with high contents in both stilbenoid DP1-2 and stilbenoid DP3-4. High capacities for ABTS and DPPH and ORAC activities were observed for grape cane extracts from Savagnin blanc, Villard noir and Magdeleine noire des Charentes. Pairwise correlations between polyphenol and antioxidant capacities were performed to identify molecules which contributed the most to the antioxidant capacities within a complex mixture of polyphenols. As a result, *E*-resveratrol (3), *E*-piceatannol (4), *E*-ε-viniferin (13), hopeaphenol (36), isohopeaphenol (37) and *Z*/*E*-vitisin B (41) were found to be the main drivers of ABTS and DPPH capacities using two antioxidant tests based on the mixed-mode (HAT/SET) antioxidant ability. The present approach might assist grape varietal selection programs to improve the human health benefit potentials of dietary grape extracts based on the wood biomass.

## Figures and Tables

**Table 1:** Antioxidant activities of polyphenol-enriched grape stems extracts using ORAC, ABTS, DPPH, FRAP, CUPRAC and iron chelation activity. ^1^ Means within a column (n = 3) followed by different letters differ significantly (Kruskal–Wallis one-way ANOVA on ranks and Fisher LSD, *p* < 0.05). ^2^ Expressed as Trolox equivalent antioxidant capacity (µmol TEAC.g^−1^ of extract). ^3^ Expressed as Ascorbic acid equivalent antioxidant capacity (µmol Ascorbic acid·g^−1^ of extract). ^4^ Expressed as fixed iron (µM·g^−1^ of extract). ^5^ Effect of the grape stems variety on the antioxidant activity (*** *p* < 0.001).

Variety	ORAC ^2^	ABTS ^2^	DPPH ^2^	FRAP ^3^	CUPRAC ^3^	Chelation ^4^
V20 Icod de los Vinos	8215 ^1^ ± 24 ^h^	12,795 ± 74 ^h^	13,070 ± 47 ^c^	14,120 ± 1762 ^de^	10,824 ± 1215 ^cd^	927 ± 64 ^c^
V21 Kecskemeti Rizling	8436 ± 45 ^g^	13,471 ± 137 ^g^	12,744 ± 170 ^d^	16,218 ± 2468 ^bc^	12,271 ± 1702 ^ab^	1077 ± 61 ^a^
V30 Piquepoul	10,172 ± 24 ^b^	12,662 ± 72 ^h^	13,179 ± 47 ^c^	15,697 ± 1550 ^bc^	11,912 ± 1069 ^bc^	1126 ± 97 ^a^
V37 Sémillon	9656 ± 283 ^c^	14,493 ± 102 ^e^	13,302 ± 170 ^bc^	16,918 ± 625 ^ab^	12,754 ± 431 ^ab^	1107 ± 85 ^a^
V40 Tsaoussi	8650 ± 24 ^f^	14,127 ± 74 ^f^	13,206 ± 295 ^c^	14,076 ± 381 ^de^	10,794 ± 263 ^cd^	1069 ± 48 ^a^
V22 Magdeleine N	8863 ± 28 ^e^	14,780 ± 87 ^cd^	13,560 ± 47 ^ab^	17,767 ± 1378 ^a^	13,340 ± 950 ^a^	1088 ± 36 ^a^
V32 Riesling	9670 ± 148 ^c^	14,596 ± 117 ^de^	13,288 ± 295 ^bc^	13,321 ± 240 ^ef^	9170 ± 1106 ^e^	1048 ± 43 ^ab^
V34 Sauvignon	10,882 ± 26 ^a^	14,837 ± 80 ^bc^	13,206 ± 0 ^c^	12,578 ± 434 ^f^	8842 ± 638 ^e^	1108 ± 62 ^a^
V35 Savagnin	9776 ± 25 ^c^	15,227 ± 20 ^a^	13,234 ± 170 ^c^	18,052 ± 585 ^a^	13,536 ± 403 ^a^	1093 ± 78 ^a^
V42 Villard noir	9141 ± 136 ^d^	15,020 ± 239 ^b^	13,778 ± 163 ^a^	15,331 ± 410 ^cd^	9821 ± 1066 ^de^	951 ± 41 ^bc^
*p*-value ^5^	***	***	***	***	***	***

## Data Availability

All of the data supporting the findings of this study are included in this article.

## Supplementary Materials

The following supporting information can be downloaded at: https://www.mdpi.com/article/10.3390/molecules27134029/s1, Table S1: list of grape stem polyphenols identified in studied cultivars, Figure S1: variability of single compound within a pool of 44 European varieties. Each compound is colored according to its phenolic class.

## Sample Availability

Samples are available from the authors.
