# Supplementary material for: Identifying Major Drivers of Antioxidant Activities in Complex Polyphenol Mixtures from Grape Canes
Source: Molecules. 2022 Jun 23;27(13):4029. doi: 10.3390/molecules27134029 (PMC9268674; doi:10.3390/molecules27134029)
Supplement: Supplementary file 1 [file molecules-27-04029-s001.zip › molecules-1778056-SM.pdf]

molecules

MDPI

Table S1. List of grape stem polyphenols identified in studied cultivars. * identified compound annotation from literature data.

Peak	RT (min)	Phenolic Class	Compound Assignment	*m/z* [M − H]⁻	Product Ions (ES−)	*m/z* [M + H]⁺	λmax (nm)	References
1	1.69	Phenolic acid	gallic acid	169	163, 125		269	Standard
2	3.58	Phenolic acid	caffeic acid	179	135		324	Standard
3	9.65	Stilbenoid DP1	*E*-resveratrol	227	183, 143	229	305, 317	Standard
4	9.17	Stilbenoid DP1	*E*-piceatannol	243	185, 159	245	322	Standard
5	5.38	Flavonoid	catechin	289	261, 205	291	229, 278	Standard
6	6.73	Flavonoid	epicatechin	289	211, 152	291	229, 278	Standard
7	4.12	Flavonoid	gallocatechin	305	109	307	332, 369	Standard
8	8.43	Stilbenoid DP1	*E*-piceid	389	227		278.7	Standard
9	8.48	Flavonoid	epicatechin 3-*O*-gallate*	441	289, 169	443	276.7	[4]
10	9.09	Flavonoid	astilbin	449	303, 285		231, 289.7	Standard
11	10.22	Stilbenoid DP2	pallidol	453	277, 265	455	232sh, 279, 285	Standard
12	13.39	Stilbenoid DP2	*Z*-resveratrol dimer*	453	428	455	225, 282.7	[5]
13	13.73	Stilbenoid DP2	*E*-ε-viniferin	453	411, 369	455	225sh, 323	Standard
14	14.81	Stilbenoid DP2	*E*-ω-viniferin*	453	369, 263	455	225sh, 323	[5]
15	15.36	Stilbenoid DP2	*E*-δ-viniferin	453	227	455	225sh, 309	Standard
16	8.25	Flavonoid	quercetin-3-O-glucoside	463	301	465		Standard
17	8.93	Stilbenoid DP2	ampelopsin A	469	451, 316	471	281.7	Standard
18	10.84	Stilbenoid DP2	scirpusin A1*	469	451, 395	471	324.7	[8,23]
19	12.39	Stilbenoid DP2	scirpusin A2*	469	379, 301	471	321.7	[8,23]
20	7.47	Stilbenoid DP2	restrytisolA*	471	377, 246		230, 276	[6,10,24]
21	8.32	Stilbenoid DP2	restrytisolB*	471	379, 349		235, 267	[6,10,24]
22	8.42	Stilbenoid DP2	restrytisol3*	471	389, 227		234, 330	[6,10,24]
23	8.75	Flavonoid	quercetin-3-*O*-glucuronide	477	301, 151	479	256, 354	Standard
24	4.76	Procyanidin	procyanidin B1	577	295, 162	579	280, 313	Standard
25	7.02	Procyanidin	procyanidin B2	577		579		Standard
26	5.13	Procyanidin	procyanidin B3*	577	295, 162	579	280, 313	[4]
27	6.18	Procyanidin	procyanidin B4*	577	514	579		[4]
28	10.62	Stilbenoid DP2	resveratrol dimer glycoside*	615	567, 537		323	[8]
29	14.71	Stilbenoid DP3	α-viniferin*	677	423	679	230sh, 285	[11]
30	12.34	Stilbenoid DP3	resveratrol trimer1*	679		681		[5,8]
31	12.91	Stilbenoid DP3	resveratrol trimer2*	679	383			[5,8]
32	14.06	Stilbenoid DP3	*E*-miyabenol C	679	573, 345	681	284	Standard
33	15.31	Stilbenoid DP3	resveratrol trimer3*	679	653, 454	681	230sh, 322	[5,8]
34	5.63	Procyanidin	procyanidin trimer*	865	664, 576	867	279	[12]
35	14.45	Stilbenoid DP4	dehydrogenated resveratrol tetramer *	904	679		295	[13]
36	12.17	Stilbenoid DP4	hopeaphenol	905	811, 717, 705, 451, 359	907	281.6	Standard
37	12.56	Stilbenoid DP4	isohopeaphenol	905	811, 717, 451, 359	907	283.9	[25]
38	13.06	Stilbenoid DP4	resveratrol tetramer1*	905		907	285	[5,26]
39	13.56	Stilbenoid DP4	resveratrol tetramer2*	905		907	284, 331	[5,26]
40	16.03	Stilbenoid DP4	resveratrol tetramer3*	905	573	907	323	[5,26]
41	16.2	Stilbenoid DP4	*Z*/*E*-vitisin B	905	799, 359	907	322	Standard
42	12.15	Stilbenoid DP4	viniferol E*	923				[15]

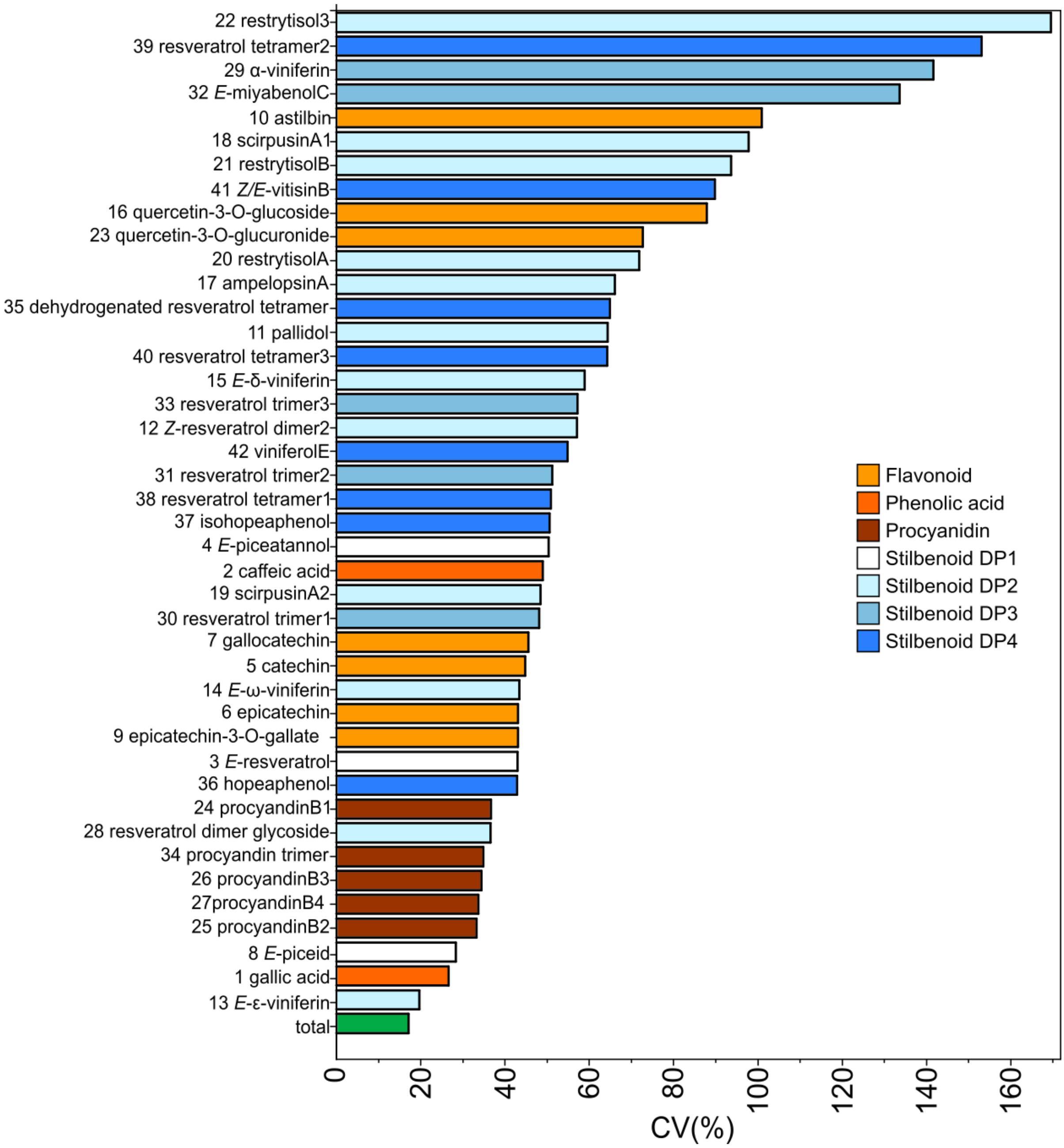

Figure S1. Variability of single compound within the pool of 44 European varieties. Each compound is colored according its phenolic class.
